# Coinfection with PEDV and BVDV induces inflammatory bowel disease pathway highly enriched in PK-15 cells

**DOI:** 10.1186/s12985-022-01845-8

**Published:** 2022-07-16

**Authors:** Jinghua Cheng, Jie Tao, Benqiang Li, Ying Shi, Huili Liu

**Affiliations:** 1 Institute of Animal Science and Veterinary Medicine, Shanghai Academy of Agricultural Science, No. 2901 Beidi Road, Minhang District, Shanghai, 201106 People’s Republic of China; 2 Shanghai Key Laboratory of Agricultural Genetic Breeding, Shanghai, 201106 People’s Republic of China; 3 Shanghai Engineering Research Center of Pig Breeding, Shanghai, 201302 People’s Republic of China

**Keywords:** Porcine epidemic diarrhea virus, Bovine viral diarrhea virus, Coinfection, Proteomics, Pathway analysis

## Abstract

**Background:**

From the 1078 diarrhea stools tested in our survey from 2017 to 2020 in local area of China, PEDV was the key pathogen that was closely related to the death of piglets with diarrhea. In addition, coinfection of PEDV-positive samples with BVDV reached 17.24%. Although BVDV infection in swine is typically subclinical, the effect of PEDV and BVDV coinfection on disease severity and the potential molecular mechanism of coinfection with these two viruses remain unknown.

**Methods:**

In this study, we developed a model of coinfection with porcine epidemic diarrhea virus (PEDV) and bovine viral diarrhea virus (BVDV) in PK15 cells, and a tandem mass tag (TMT) combined with LC–MS/MS proteomic approach was used to identify differential protein expression profiles. Additionally, we performed drug experiments to explore the inflammatory response induced by PEDV or BVDV mono- or coinfection.

**Results:**

A total of 1094, 1538, and 1482 differentially expressed proteins (DEPs) were identified upon PEDV monoinfection, BVDV monoinfection and PEDV/BVDV coinfection, respectively. KEGG pathway analysis revealed that PEDV and BVDV coinfection led to a highly significantly enrichment of the inflammatory bowel disease (IBD) pathway. In addition, the NF-κB signaling pathway was more intensively activated by PEDV and BVDV coinfection, which induced higher production of inflammatory cytokines, than PEDV or BVDV monoinfection.

**Conclusions:**

Our study indicated that cattle pathogens might play synergistic roles in the pathogenesis of porcine diarrhea, which might also improve our understanding of the pathogenesis of multiple infections in diarrhea.

**Supplementary Information:**

The online version contains supplementary material available at 10.1186/s12985-022-01845-8.

## Introduction

Viral diarrhea causes high morbidity and mortality among pigs, leading to large economic losses in the swine industry. It has been reported that airborne porcine epidemic diarrhea virus (PEDV) has higher transmissibility than other seasonal diarrhea viruses [1]. As a member of the Coronaviridae family, PEDV is an enveloped, single-stranded positive-sense RNA virus [2]. It mainly infects the epithelial cells of the porcine small intestine, leading to acute watery diarrhea, vomiting, and dehydration [3]. Epidemiological investigations have shown that piglet diarrhea is often caused by mixed infections and that PEDV infections are often accompanied by other diarrhea viruses, such as transmissible gastroenteritis virus (TGEV), porcine rotavirus (PoRV), porcine deltacoronavirus (PDCoV), and porcine astrovirus (PAstV) [4–6]. Besides, bovine viral diarrhea virus (BVDV), a cattle pathogen, could be detected in suckling piglet diarrhea samples [7, 8]. Undoubtedly, multiple infections with two or even more viruses that cause piglet diarrhea are quite common in clinical cases and pose a problem for the diagnosis and treatment of the disease [9, 10].

BVDV is primarily the pathogen of cattle, but other animals, such as pigs, are also susceptible [11]. From the 1078 diarrhea stools tested in our survey from 2017 to 2020 in local area of China, PEDV was the key pathogen that was closely related to the death of diarrhea piglets. In addition, coinfection of PEDV-positive samples with BVDV reached 17.24% (unpublished data). This indicated that cattle pathogens might play synergistic roles in the pathogenesis of porcine diarrhea. Although BVDV infection in swine is typically subclinical, the effect of PEDV and BVDV coinfection on disease severity and the potential molecular mechanism of coinfection with these two viruses remain unknown. Therefore, we aimed to establish a reliable system in vitro to investigate the cellular responses to PEDV and BVDV coinfection, which may increase our understanding of the host response to viral coinfection and highlight potential targets for the development of antiviral agents.

Proteomics techniques are effective tools for discovering new responsive proteins and molecular interactions under different conditions. Recently, quantitative proteomic and bioinformatic analyses have been used to accurately identify changes in protein profiles and host responses involved in viral infection. For example, in TGEV-infected cells, the iTRAQ (isobaric tags for relative and absolute quantification)-based quantitative proteomic method has been used to identify differentially expressed proteins (DEPs), and upregulated proteins were shown to be associated with interferon signaling [12]. In duck reovirus-infected liver cells, DEPs were quantified by TMT-labeled quantitative proteomic analysis, and most of the metabolism-related proteins were downregulated, suggesting a decrease in basal metabolism under viral infection [13]. Proteomic analysis was also used in the study of different viral coinfections. Zhou et al. conducted three independent comparative proteomic experiments of PCV2-CSFV mono- and coinfected cells to explore host cell responses and demonstrated that PCV2 played the dominant role in PCV2-CSFV-coinfected cells [14]. Shrinet et al. studied perturbations in the proteome of Aedes mosquitoes upon mono- and coinfection with CHIKV and DENV and revealed significantly regulated pathways [15]. These studies have outlined the dynamic interactions between host and pathogen and promote a better understanding of the pathogenesis of viral infections.

In this study, a quantitative proteomics approach based on TMT combined with LC–MS/MS was used to identify differential protein expression profiles of cells monoinfected or coinfected with PEDV/BVDV. DEPs were identified and classified into various signaling pathways by bioinformatic analyses. Importantly, we observed that the inflammatory bowel disease (IBD) pathway was highly significantly enriched under PEDV and BVDV coinfection. Moreover, coinfection with the two viruses induced stronger inflammatory cytokine production and NF-κB activity than monoinfection. Overall, this is the first study comparing the whole protein profiles of cells monoinfected with PEDV or BVDV and cells coinfected with PEDV/BVDV by quantitative proteomics. Our study indicated that cattle pathogens might play synergistic roles in the pathogenesis of porcine diarrhea, which might also improve our understanding of the pathogenesis of multiple infections in diarrhea.

## Materials and methods

### Cells, viruses, reagents and antibodies

Porcine kidney (PK15) cells were purchased from ATCC (Manassas, VA, USA) and cultured in antibiotic-free DMEM (Gibco, Grand Island, NY, USA) supplemented with 10% fetal bovine serum (FBS) (Gibco) at 37 °C in a humidified atmosphere containing 5% CO_2_. The PEDV strain JS-2/2014 was isolated from a piglet with watery diarrhea and stored in our laboratory. The BVDV-2 strain SH-28 was kindly provided by Prof. Guoqiang Zhu of Yang Zhou University. The NF-κB inhibitor (BAY11-7082) was purchased from Selleck (Houston, TX, USA). Antibodies against IκBα, p-IκBα, GAPDH and β-actin were purchased from Cell Signaling Technology (Danvers, MA, USA). Polyclonal antibody against BVDV E2 protein was purchased from Bioss Biotechnology (Beijing, China). The monoclonal antibody directed against the PEDV N protein was purchased from BioNote (Hwaseong-si, South Korea).

### Infection and coinfection of PK15 cells with PEDV and BVDV

PK15 cells were cultured in 6-well dishes to approximately 80% confluence. Then, the cells were monoinfected with PEDV strain JS-2/2014 or BVDV-2 strain SH-28 or coinfected with both strains at a multiplicity of infection (MOI) of 0.01 and incubated in serum-free DMEM containing 2 µg/mL trypsin (Promega, Madison, WI, USA). Uninfected cells served as the mock-infected group. Viral propagation was confirmed by indirect immunofluorescence assay (IFA) and Western Blot assay.

### IFA for the detection of PEDV and BVDV coinfection in PK15 cells

PK15 cells grown on a 6-well plate were infected with PEDV or BVDV or coinfected with both at 0.01 MOI. At 6, 12, and 24 h post infection (h.p.i.), the cells were fixed in 4% paraformaldehyde, permeabilized with 0.5% Triton X-100 for 10 min, and incubated in blocking buffer. IFA was then performed by staining with a monoclonal antibody against PEDV N protein and a polyclonal antibody against BVDV E2 protein. After gentle washing, the cells were incubated with FITC-conjugated donkey anti-mouse IgG and Alexa Fluor 546-conjugated donkey anti-rabbit IgG (Thermo Fisher Scientific, Waltham, MA, USA) as secondary antibodies. The cell nuclei were stained with 4′,6′-diamidino-2-phenylindole (DAPI) (Beyotime Biotechnology, Shanghai, China). The cells were visualized under a florescence microscope (Carl Zeiss, Oberkochen, Germany).

### Titer determination of virus stocks

PK15 cells grown on 35-mm dishes were infected with PEDV or BVDV or coinfected with both at 0.01 MOI. After 1 h, the medium was replaced with fresh DMEM containing 2 µg/mL trypsin. Cultured samples were collected at 24, 48, 72, and 96 h.p.i. for TCID_50_ determination by IFA. The IFA protocol was as described above. Virus titers were determined by viewing the infected cells under a fluorescence microscope and calculated on the basis of the Reed–Muench method.

### Western Blot assay

The infected cells were harvested at the indicated time points and lysed in RIPA buffer (Beyotime). Equal amounts of total proteins were separated on SDS–PAGE gels. After electrophoresis, proteins were transferred onto nitrocellulose filter membranes (Millipore, Billerica, MA) and stained with primary antibodies overnight at 4 °C, followed by HRP-conjugated secondary antibodies at room temperature for 1 h. The protein bands were detected using enhanced chemiluminescence detection kits (Thermo Fisher Scientific, Waltham, MA, USA).

### Sample preparation for proteomics, protein isolation, labeling with TMT reagents, and LC–MS/MS analysis

PK15 cells were monoinfected with PEDV strain JS-2/2014 or BVDV strain SH-28 or coinfected with both at an MOI of 1. At 24 h.p.i., cells from all experimental groups were collected, washed three times with ice-cold PBS, and lysed with 1 mL lysis buffer (8 M urea, 50 mM Tris–HCl, 0.2% SDS, pH 8.5) followed by ultrasonication on ice for 5 min. The lysate was centrifuged at 12,000 × g for 10 min to remove the insoluble debris. The supernatant was collected and reduced with 2 mM DTT at 56 °C for 1 h and subsequently alkylated with sufficient iodoacetamide for 1 h. The extracted proteins were precipitated with precooled acetone, washed twice, and redissolved in buffer containing 0.1 M TEAB and 8 M urea (pH 8.5). The protein concentration was quantified by the Bradford protein assay. A total of 100 µg protein from each sample was digested with Trypsin Gold (Promega). After trypsin digestion, peptides were desalted using a C18 cartridge to remove urea and dried by vacuum centrifugation. For TMT labeling, the peptides were processed using 6-plex TMT reagents according to the protocol (Thermo Fisher Scientific). The labeled peptides were fractionated using a C18 column on a Rigol L3000 HPLC. The samples were then dried using a vacuum and reconstituted in 0.1% (v/v) formic acid (FA) in water for subsequent LC–MS/MS analysis. This analysis was performed by Novogene Bioinformatics Technology Co., Ltd.

### Proteomics data normalization and analysis

Spectral data were processed using Proteome Discoverer 2.2 with the MASCOT engine (version 2.2; Matrix Science, London, UK) against the UniProt database (Sus_scrofa_uniprot_2020_1_8.fasta), containing 120,594 sequences. The mass error was set to 10 ppm for precursor ions and to 0.02 Da for fragment ions, and a maximum of 2 miscleavage sites were allowed. For protein quantitation, proteins were required to contain at least 1 unique peptide and a false discovery rate (FDR) of no more than 1.0%. Statistical analyses of data among groups were performed using Student's t test. For accurate comparisons between samples, proteins with fold changes ≥ 1.2 or ≤ 0.83 and a *P value* < 0.05 were considered as DEPs.

### Bioinformatic analysis

Functional classification of DEPs was performed based on Gene Ontology (GO) enrichment analysis (http://www.geneontology.org/). Pathway enrichment analysis of DEPs was carried out using the Kyoto Encyclopedia of Genes and Genomes (KEGG) database (http://www.genome.jp/kegg/). Enriched KEGG pathways with Bonferroni adjusted *P* value (*q* value) < 0.05 were considered significant by using the hypergeometric test.

### Relative quantitative PCR (qPCR)

Total RNA was extracted from infected and uninfected cells at the indicated times using TRIzol reagent (Thermo Fisher Scientific). cDNA synthesis was performed using the HiScript II 1st Strand cDNA Synthesis Kit (Vazyme Biotech, Nanjing, China) according to the manufacturer’s protocol. qPCR was carried out using SYBR Premix Ex Taq II (TaKaRa Bio, Shiga, Japan) with an ABI 7500 sequence detection system (ABI, Madison, USA). The amplification conditions consisted of 95 °C for 30 s and 40 cycles of 95 °C for 5 s, 60 °C for 30 s, and 95 °C for 15 s. The β-actin gene was used as an internal standard, and the relative expression levels of each gene in the samples were calculated using the 2^−ΔΔCt^ method [16]. The primer sequences used for amplifications were as follows: IL-6 (F: ATTCGGTACATCCTC GACGGC, R: CAGCCATCTTTGGAAGGTTCAGGT), IL-8 (F: TTTCAGAGACAGCAGAGCACA, R: CACACAGAGCTGCAGAAATCAG), IL-18 (F: GAATCTAAATTATCAGTCATAAG, R: GATAGATCTATAATGTTCACTG), TNF-α (F: CTCAGCAAGGACAGCAGAGG, R: ATGTGGCGTCTGAGGGTTGTT), and β-actin (F: TGGGTCAGAAGGACTCCTATG, R: CAGGCAGCTCATAGCTCTTCT).

### Luciferase reporter gene assays

PK15 cells were cultured in 24-well plates and cotransfected with 100 ng of the luciferase reporter pNF-κB-luc and 10 ng of the constitutive Renilla luciferase reporter pRL-TK (Promega). After transfection for 24 h, the cells were infected with PEDV or BVDV or coinfected with both at an MOI of 1. Then, 24 h later, the cells were lysed and subjected to luciferase assays using the Dual Luciferase Reporter Assay System (Promega) according to the manufacturer’s protocol. The results are shown as the means ± SD of triplicate wells and expressed as relative luminescence units (RLUs).

### Drug treatment

Drug experiment was conducted to inhibit the NF-κB pathway. PK15 cells were treated with 1 μM Bay11-7082 or DMSO as a control, and monoinfected with PEDV strain JS-2/2014 or BVDV strain SH-28 or coinfected with both at an MOI of 1. At 1 h of virus infection, the cells were washed, and the medium was replaced with maintenance medium containing Bay11-7082 at 1 μM. Then, 24 h later, the cells were subjected to qPCR analyses of inflammatory cytokine expression.

## Results

### BVDV and PEDV can productively infect PK15 cells

**Fig. 1:**
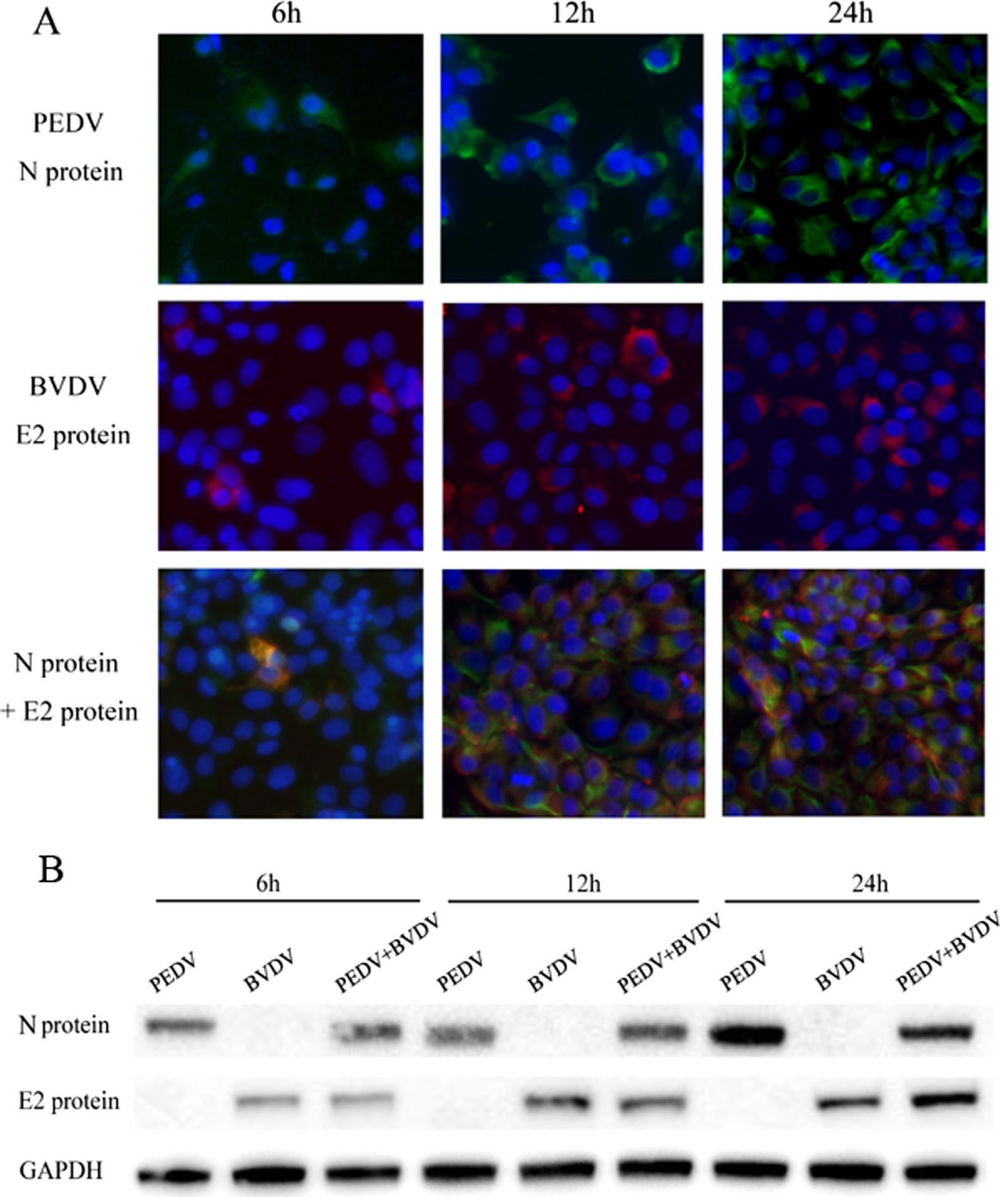
PEDV and BVDV coinfection in PK15 cells. PK15 cells were monoinfected with PEDV strain JS-2/2014 or BVDV strain SH-28 or coinfected with both at an MOI of 0.01. Cells were collected at 6, 12, and 24 h.p.i. **A** The cells were fixed and stained for IFA. Green, PEDV N protein; red, BVDV E2 protein; blue, DAPI-stained for the nucleus. **B** The expression of PEDV N protein and BVDV E2 protein in PK15 cells was analyzed by Western Blot

Immunofluorescence assays and Western Blot were performed to determine whether PK15 cells can support BVDV and PEDV replication and viral protein synthesis. For IFA, PEDV N protein and BVDV E2 protein were stained with the corresponding antibody and visualized using a fluorescence microscope. As shown in Fig. 1A, the N and E2 proteins were mainly distributed in the cytoplasm of PK15 cells, and cells monoinfected or coinfected with PEDV and BVDV started to exhibit immunofluorescence at 6 h.p.i., with more intense signals at 12 and 24 h.p.i.. Similarly, a Western Blot assay showed that the PEDV N and BVDV E2 proteins could be detected at 6 h.p.i., and their expression was strongly increased at 12 and 24 h.p.i. The results of the Western Blot assay were consistent with the IFA results, indicating that the PEDV strain JS-2/2014 and the BVDV-2 strain SH-28 could simultaneously and effectively replicate in PK15 cells.

### Kinetics of PEDV and BVDV multiplication in PK15 cells

**Fig. 2:**
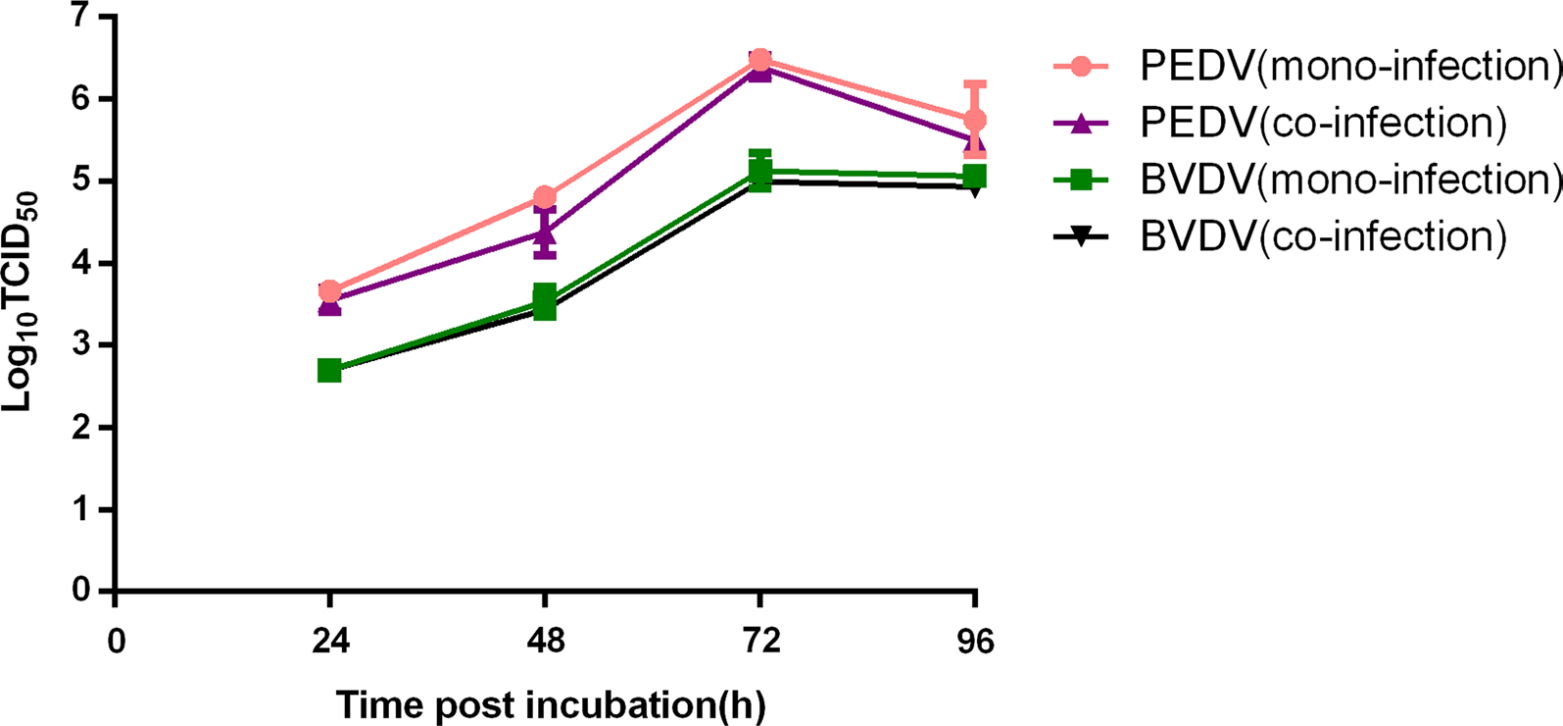
Growth curve of BVDV and PEDV in PK15 cells. PK15 cells were monoinfected with PEDV strain JS-2/2014 or BVDV strain SH-28 or coinfected with both at an MOI of 0.01. At 24, 48, 72 and 96 h.p.i., the virus titers in the supernatants were determined using a TCID_50_ assay. The mean values from three independent experiments are shown for each sample

To determine the kinetics of PEDV and BVDV propagation in PK15 cells, we infected cells with JS-2/2014, SH-28, or both at an MOI of 0.01 and monitored viral titers at different time points after infection. Virus growth curves showed that the titers of the two viruses increased gradually as the incubation time increased and peaked at 72 h.p.i. (Fig. 2). Although the viral titers of JS-2/2014 and SH-28 were slightly higher in monoinfected cells than in coinfected cells, the differences were not significant (*P* > 0.05).

### Protein profile by TMT combined with LC–MS/MS analysis

**Fig. 3:**
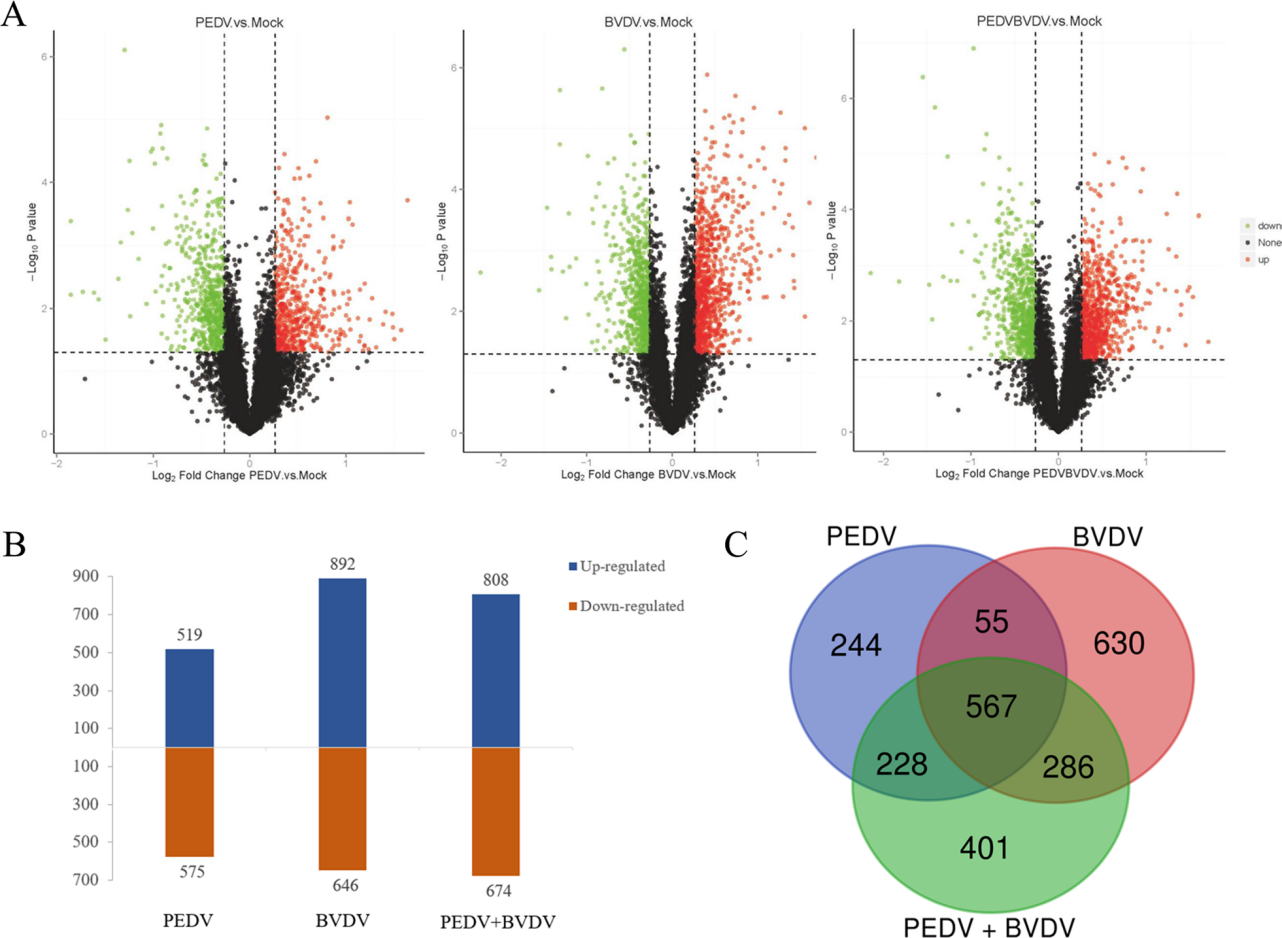
DEPs in PK15 cells. **A** Volcano plot of DEPs in cells monoinfected with PEDV, BVDV or coinfected with both, compared to noninfected cells. Fold-changes ≥ 1.20 or ≤ 0.83 and *P* < 0.05 were considered statistically significant. Red dots, significantly upregulated proteins; green dots, significantly downregulated proteins. **B** Numbers of upregulated or downregulated proteins in cells monoinfected with PEDV, BVDV or coinfected with both. **C** Venn diagram of the DEPs involved in the PEDV and BVDV monoinfection or coinfection groups

In total, 7975, 6891 and 6891 proteins were identified and quantified by TMT coupled with LC–MS/MS analysis in cells monoinfected with PEDV strain JS-2/2014 or BVDV strain SH-28 or coinfected with both, respectively. Volcano plot analysis was performed using the criteria of proteins with fold-changes ≥ 1.20 or ≤ 0.83 and *P* < 0.05 between the virus infection group and control (Fig. 3A). According to this threshold, a total of 1094, 1538 and 1482 DEPs were identified in PEDV -infected, BVDV-infected and PEDV/BVDV coinfected cells, respectively (Additional file 1: File S1). Among these DEPs, 519 were upregulated and 575 were downregulated significantly in PEDV-infected cells, and 892 were upregulated and 646 were downregulated significantly in BVDV-infected cells. In PEDV and BVDV coinfected cells, the number of protein up and downregulated proteins was 808 and 674, respectively (Fig. 3B). Furthermore, 244, 630 and 401 DEPs were found to be specific in PEDV-infected, BVDV-infected and PEDV/BVDV coinfected cells, respectively, while 567 DEPs were shared among all the infected groups (Fig. 3C).

### Bioinformatics analysis of the PK15 cell proteome

**Fig. 4:**
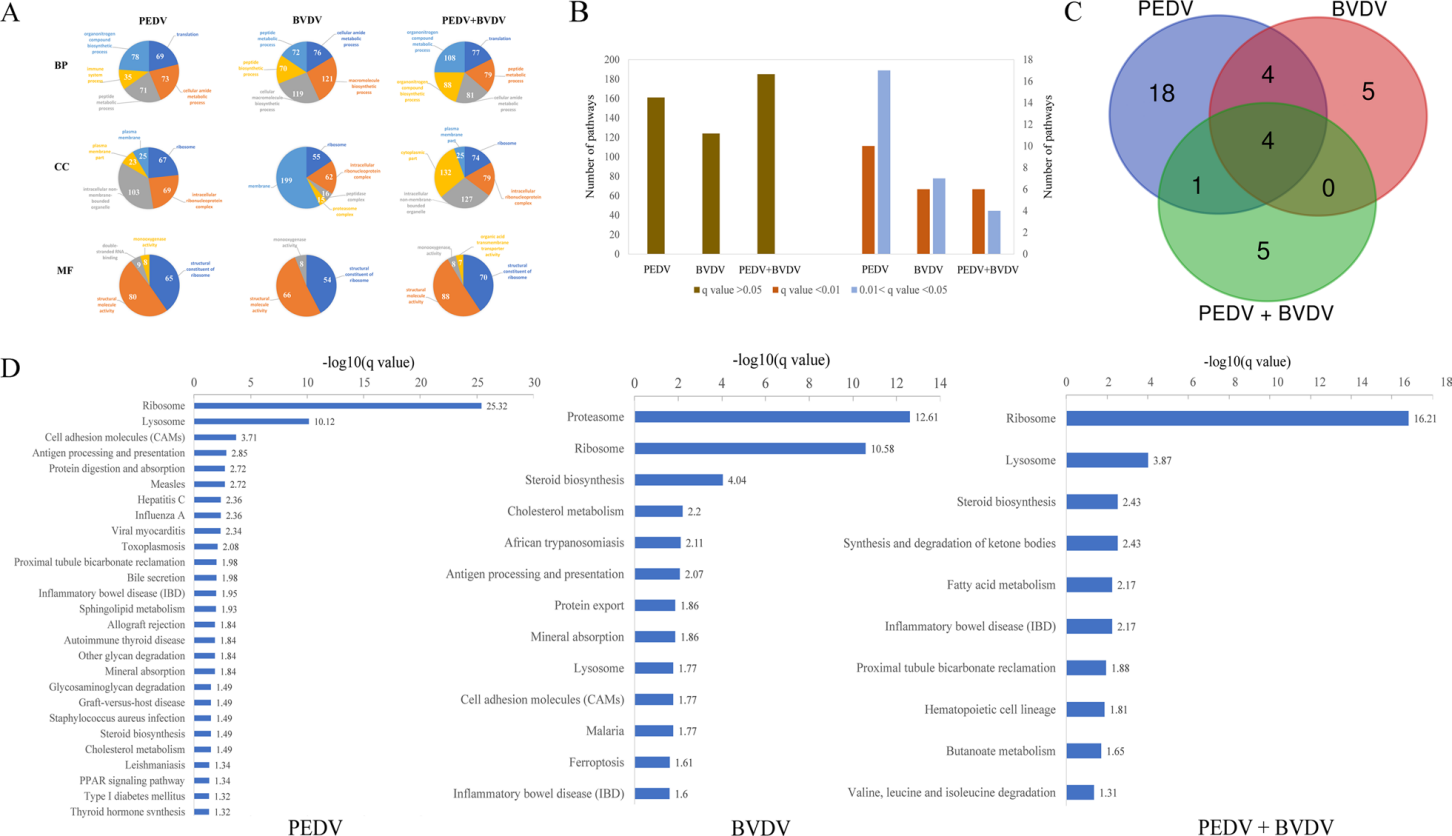
Classification and annotation of the DEPs. **A** The top five significantly enriched GO terms of ‘cellular component’, ‘biological process’ and ‘molecular function’ in the PEDV and BVDV monoinfection or coinfection groups. **B** Numbers of related KEGG pathways upon PEDV and BVDV monoinfection or coinfection. **C** Venn diagram of significantly enriched KEGG pathways of PEDV and BVDV monoinfection or coinfection groups. **D** Significantly enriched KEGG pathways of PEDV and BVDV monoinfection or coinfection groups

Based on GO enrichment analysis, all DEPs were assigned to three categories of GO terms: ‘cellular component’, ‘biological process’ and ‘molecular function’ (Additional file 2: File S2). In the biological process category, DEPs were strongly represented by the terms ‘biosynthetic process’ and ‘metabolism process’. The DEPs were also enriched in numerous cellular components, of which ‘ribosome, intracellular ribonucleoprotein complex’ and ‘intracellular nonmembrane-bounded organelle’ were the main ones, and in molecular functions, of which ‘structural molecule activity’ and ‘structural constituent of ribosome’ were dominant. In Fig. 4A, we present the top five significantly enriched of ‘cellular component’, ‘biological process’ and ‘molecular function’ GO terms in the PEDV and BVDV monoinfection and coinfection groups. Overall, the GO annotation comparison could provide a comprehensive overview of the molecular characterization of PEDV and BVDV monoinfection and coinfection.

To compare the differences in enriched metabolic pathways, KEGG pathway enrichment analysis was performed to elucidate the DEPs identified in cells monoinfected or coinfected with PEDV and BVDV. As shown in Fig. 4B, 27, 13 and 10 pathways were significantly enriched in the PEDV monoinfection, BVDV monoinfection and PEDV /BVDV coinfection groups, respectively. A Venn diagram showed that a total of 37 pathways were significantly enriched in the PEDV and BVDV mono- or coinfection groups, 4 of which were shared among the three groups (Fig. 4C). The significantly enriched KEGG pathways of PEDV and BVDV monoinfection or coinfection groups are shown in Fig. 4D and Additional file 3: File S3. Notably, the IBD pathway was significantly enriched in the JS-2/2014 and SH-28 monoinfection groups, and it was highly significantly (*q* value = 0.0068, < 0.01) enriched in the coinfection group.

### Coinfection with PEDV and BVDV leads to higher levels of inflammatory cytokines

**Fig. 5:**
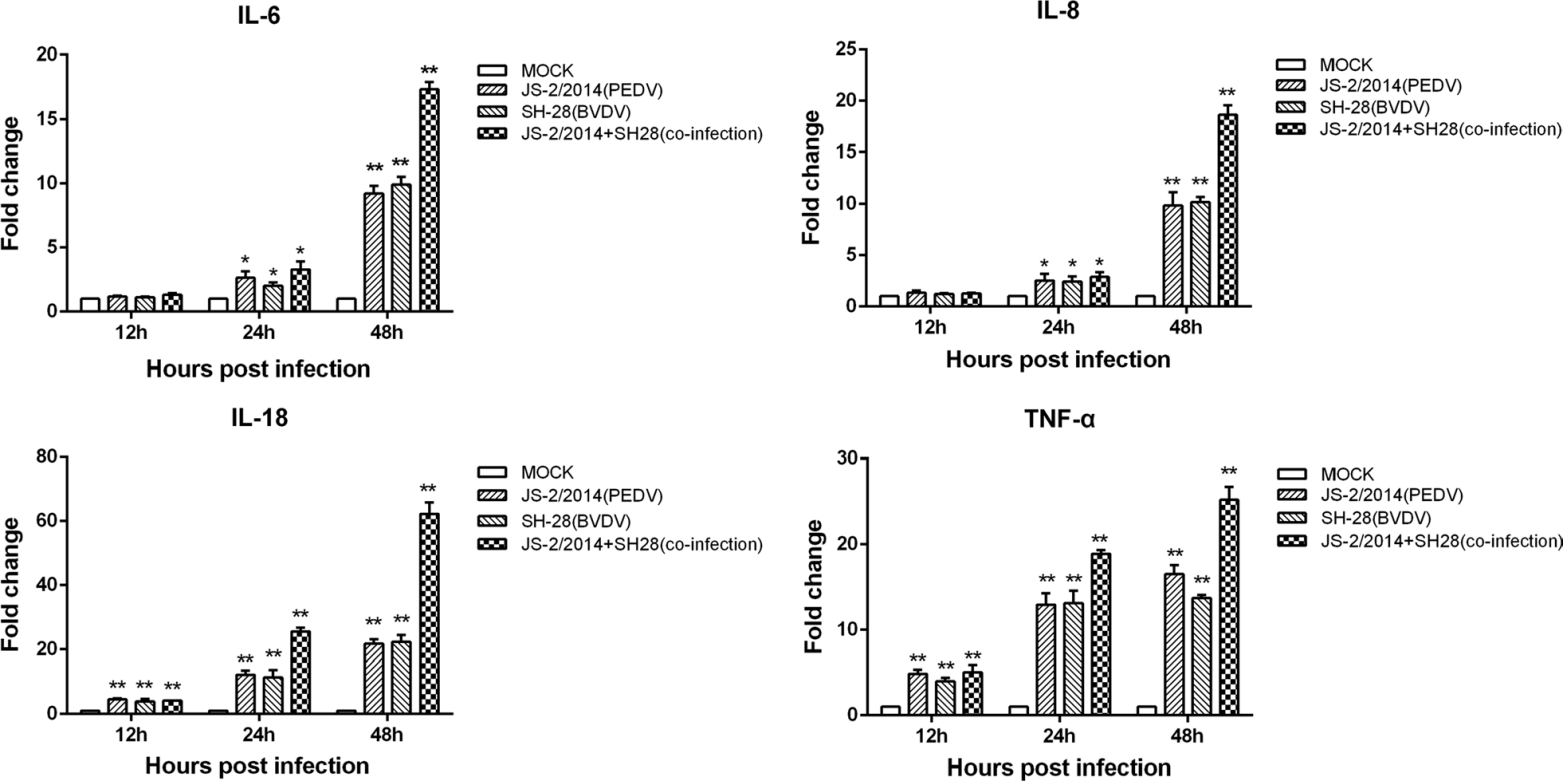
Expression of inflammatory cytokines in cells upon PEDV or BVDV monoinfection or coinfection. PK15 cells were monoinfected with PEDV, or BVDV or coinfected with both at an MOI of 1 for the indicated times, and mRNA levels of IL-6, IL-8, IL-18 and TNF-α were quantified by qPCR. The fold changes in mRNA expression levels were calculated through the comparative CT method. The error bars represent the SD of the means from three independent experiments. (*p < 0.05, **p < 0.01)

The above KEGG pathway analysis showed that the IBD pathway was enriched by virus infection, and it was highly significantly enriched by PEDV and BVDV coinfection. To test the hypothesis that coinfection with PEDV and BVDV induces higher inflammatory cytokine levels, we measured the mRNA levels of IL-6, IL-8, IL-18 and TNF-α in PK15 cells within 48 h following PEDV and BVDV monoinfection or coinfection. It was found that viral infection of PK15 cells induced inflammatory cytokine production, which continued to increase gradually over time. Compared to the control group, all virus-infected groups had significantly increased IL-18 and TNF-α mRNA at 12, 24 and 48 h.p.i. as well as increased IL-6 and IL-8 mRNA at 24 and 48 h.p.i. (Fig. 5). Additionally, coinfection with PEDV and BVDV induced higher mRNA levels of these inflammatory cytokines and caused the highest increase in IL-18 production. The results of qPCR showed that the mRNA expression of IL-18 was upregulated approximately 22-fold upon PEDV and BVDV monoinfection and 62.21-fold upon coinfection with the two viruses at 48 h.p.i.. These results revealed that inflammatory responses were induced during PEDV and BVDV infection and that the increased production of inflammatory cytokines may be related to increased severity of IBD.

### The NF-κB signaling pathway is required for cytokine expression

**Fig. 6:**
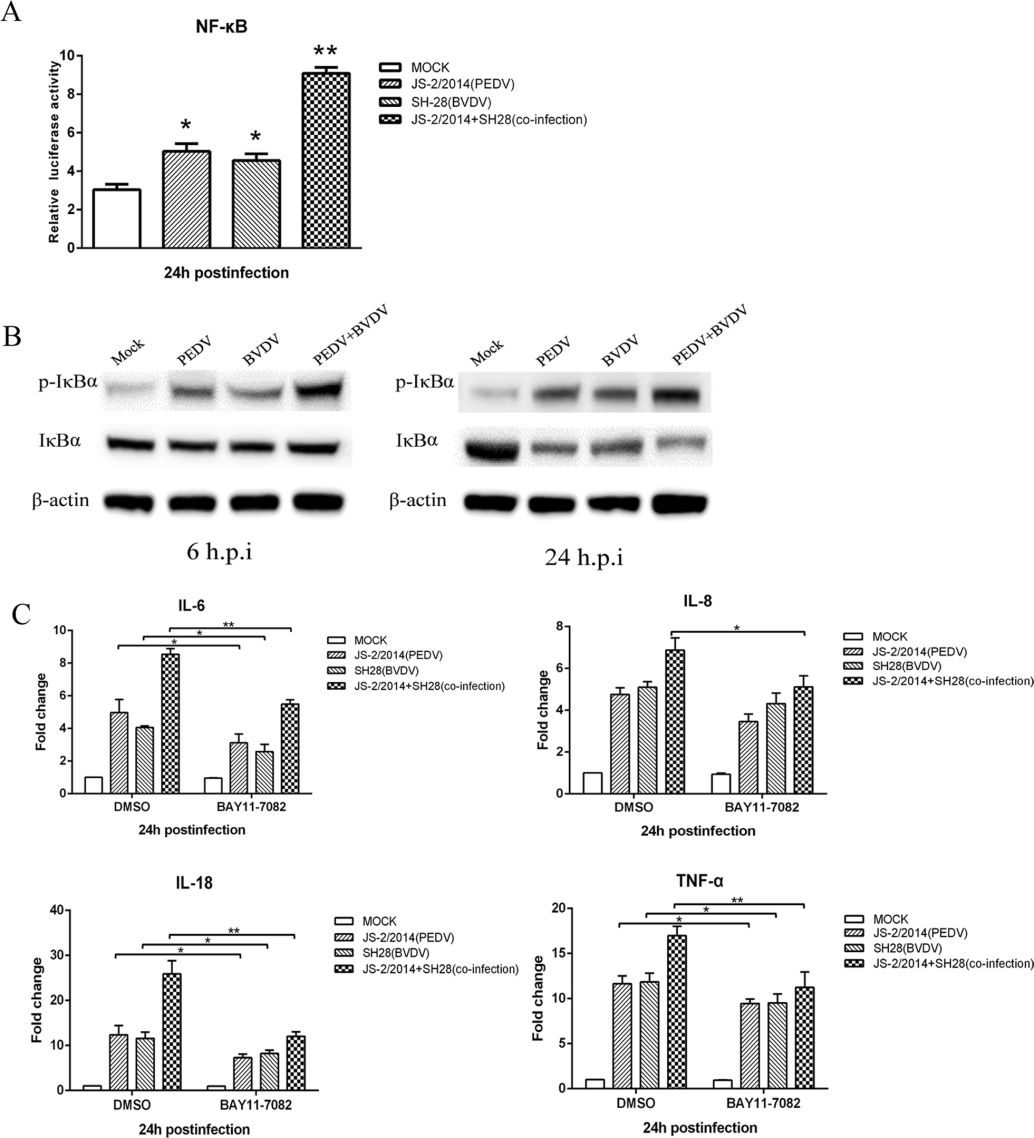
The NF-κB signaling pathway is required for cytokine production induced by PEDV/BVDV mono- or coinfection. **A** PK15 cells were cotransfected with pNFκB-luc (100 ng) and pRL-TK (10 ng). At 24 h.p.i., cells were monoinfected with PEDV strain JS-2/2014 or BVDV strain SH-28 or coinfected with both at an MOI of 1 and then harvested for luciferase activity analysis at 24 h.p.i.. **B** PK15 cells were monoinfected with PEDV strain JS-2/2014 or BVDV strain SH-28 or coinfected with both at an MOI of 1. At 6 and 24 h.p.i., the cells were harvested to detect p-IκBα and IκBα by Western Blot analysis. **C** PK15 cells were pretreated with 1 μM BAY 11-7082 (NF-κB inhibitor) or DMSO as control, and then monoinfected with PEDV strain JS-2/2014 or BVDV strain SH-28 or coinfected with both at an MOI of 1. Cells were collected at 24 h.p.i. for qPCR to analyze the relative expression of the target genes. The error bars represent the SD of the means from three independent experiments. (*p < 0.05, **p < 0.01)

NF-κB has been identified as a key regulator in the production of inflammatory cytokines and has been implicated in the pathogenesis of IBD. Therefore, we assessed the effect of viral infection on NF-κB promoter activity. The results indicated that the activity of the NF-κB promoter was enhanced by viral infection, and the effect was more obvious in coinfected cells (Fig. 6A). To determine whether the NF-κB pathway is involved in virus-induced cytokine production, PK15 cells were treated with an NF-κB pathway inhibitor (BAY11-7082) before viral infection. As shown in Fig. 6B, PEDV and BVDV coinfection significantly inhibited the mRNA levels of IL-6, IL-8, IL-18 and TNF-α by 35%, 26%, 53% and 34%, respectively, at 24 h.p.i.. The inhibition of these cytokines could also be found in cells infected with only a single virus, but the effect was not as strong as that of coinfection. To further investigate whether the NF-κB signaling pathway was more strongly activated in coinfected cells, IκBα phosphorylation and degradation of total IκBα were analyzed by Western Blot. As shown in Fig. 6C, PEDV and BVDV coinfection led to relatively stronger IκBα phosphorylation at 12 h.p.i. and 24 h.p.i., while infection with only one virus resulted in limited IκBα phosphorylation. Moreover, IκBα was gradually degraded in the later stages of infection. Overall, these results suggest that the NF-κB pathway is involved in cytokine production upon PEDV or BVDV infection and that coinfection with the two viruses induces stronger activation of the NF-κB pathway.

## Discussion

Coinfection with multiple pathogens may increase disease severity and has become a considerable concern, but the specific mechanisms of viral coinfection are not fully understood, and models for viruses coinfecting a single cell are rare. In this study, PK15 cells were chosen as host cells to establish a model of BVDV and PEDV coinfection, and Western Blot and immunofluorescence were used to track their propagation. The results showed that PEDV N protein and BVDV E2 protein were mainly distributed in the cytoplasm of PK15 cells and exhibited low levels of immunofluorescence at 12 h and more intense signals after 24 and 48 h. The Western Blot results also showed similar trends. The results above showed the colocalization of BVDV E2 and PEDV N protein in coinfected cells and indicated that these two viruses could grow stably in PK15 cells.

In the case of coinfection, the replication efficiency of the virus may be affected by the other virus, or the virus could replicate well in cells regardless of the presence or absence of the other virus [17]. To study the interactions between HBV and HCV, Bellecave established a model system and found that HBV and HCV could replicate well without overt interference in Huh-7 cell lines [18]. In a study of postweaning multisystemic wasting syndrome (PMWS) of nursery and fattening pigs, Rovira proposed that PRRSV infection enhances PCV2 replication, as demonstrated by the TaqMan PCR method [19]. According to Zhou et al.’s report, the replication cycle of PCV2 was completed without interference or decreased by CSFV infection in cells coinfected with PCV2 and CSFV; however, as the PCV2 inoculum increased, the titers and genomic copies of CSFV progeny stocks decreased gradually [20]. In our study, titers of PEDV and BVDV progeny stocks were slightly lower in coinfected cells, although no significant difference was found between coinfected and monoinfected cells. We speculated that this may result from higher levels of cytokines induced by coinfection with the two viruses that would to some extent inhibit viral replication.

To date, proteomic technology has been widely used in studying changes in global protein profiles during viral infection. Previous studies used proteomics methods to investigate protein alterations upon PEDV monoinfection, which might elucidate the pathogenic mechanisms and host response involved in PEDV infections [21, 22]. However, little information on the protein profile of coinfection with PEDV and other diarrhea pathogens is available. Here, an integrated approach involving TMT labeling combined with LC–MS/MS was applied to explore the global proteome characteristics under PEDV/BVDV monoinfection or coinfection. In this study, 1094 and 1538 differentially regulated proteins were identified in PK15 cells monoinfected with PEDV strain JS-2/2014 and BVDV strain SH-28, respectively, and 1482 DEPs were identified in coinfected cells. On the basis of proteins being significantly modulated and the pathways associated with those proteins identified by KEGG pathway enrichment analysis, PEDV and BVDV coinfection induced more profound responses to the IBD pathway.

It is thought that IBD results from an aberrant and continuing immune response to microbes in the gut, and we measured inflammatory cytokine production in cells following PEDV and BVDV monoinfection or coinfection [23]. The results indicated that IL-6, IL-8, IL-18 and TNF-α were all upregulated during PEDV and BVDV monoinfection or coinfection and were expressed at higher levels in coinfected cells. The pathogenesis of IBD is not completely clear, but the normal balance between inflammatory and regulatory cytokines is disturbed. Furthermore, we found that IL-18 was most significantly upregulated in the coinfected group. Previous studies revealed the importance of IL-18 in the pathogenesis of IBD [24]. As seen in Timna et al.’s study, serum concentrations of IL-18 are higher in patients with IBD than in healthy individuals [25]. According to a previous report, increased IL-18 transcripts and the overexpression of mature IL-18 protein were found in patients with CD, a form of IBD that is a typical Th1-mediated disease [26]. The role of IL-18 in intestinal disease is largely related to its activity in regulating proinflammatory responses. In addition, IL-18 can promote the inflammatory cascade by enhancing the release of TNF-a, IL-8, and IL-1 [27].

The NF-κB signaling pathway is involved in regulating the transcription of multiple cytokines related to inflammatory responses. It was found that the activity of NF-κB signaling was linked with the virulence and pathogenicity of PEDV, and a more dramatic activation of NF-κB signaling caused more severe inflammatory cascades to aggravate cell destruction [21]. Although both BVDV-1 and BVDV-2 infections can increase NF-κB activity, BVDV-1 infection was shown to modulate cytokine transcription and production mainly through the NF-κB pathway, while BVDV-2 infection was proven to be induced through an NF-κB-independent pathway [28]. Our results suggested synergistic effects of PEDV and BVDV coinfection on the NF-κB signaling pathway, as this pathway was more significantly activated by PEDV and BVDV coinfection. In addition, the IL-6, IL-8, IL-18 and TNF-α production induced by PEDV or BVDV mono- or coinfection was impaired to varying degrees by the NF-κB pathway inhibitor, suggesting that the production of these inflammatory cytokines is dependent on the NF-κB pathway. NF-κB also plays a central role in the pathogenesis and development of IBD. High levels of NF-κB could be observed in the mucosal cells of IBD patients, especially macrophages, and epithelial cells isolated from inflamed gut specimens from IBD patients showed augmented levels of NF-κB [29, 30]. Our results showed that the IBD pathway was highly significantly (*q* value = 0.0068, < 0.01) enriched by PEDV and BVDV coinfection, which also confirmed that the IBD pathway is positively correlated with the activation of NF-κB.

## Conclusions

In summary, we have developed a model of BVDV and PEDV coinfection, and the proteomic changes in PK15 cells coinfected with PEDV/BVDV were characterized using TMT labeling combined with LC–MS/MS. GO and KEGG pathway analyses revealed that the IBD pathway was highly significantly enriched by PEDV and BVDV coinfection. We also demonstrated that PEDV and BVDV coinfection could activate the NF-κB signaling pathway more intensively, which induced higher production of inflammatory cytokines. While the specific molecular mechanisms of viral coinfection that increase disease severity are still not fully understood, immune modulation by the coinfecting viruses likely plays an important role.

## Supplementary Information

**Additional file 1.** The fold changes of all significantly differently expressed proteins in PEDV-infected, BVDV-infected and PEDV/BVDV coinfected cells.

**Additional file 2.** Gene Ontology analysis of significantly differently expressed proteins in PEDV-infected, BVDV-infected and PEDVBVDV coinfected cells.

**Additional file 3.** KEGG enrichment analysis of significantly differently expressed proteins in PEDV-infected, BVDV-infected and PEDVBVDV coinfected cells.

## Data Availability

All data generated or analyzed during this study are available from the corresponding author on reasonable request.

## Abbreviations

PEDV: Porcine epidemic diarrhea virus
BVDV: Bovine viral diarrhea virus
TMT: Tandem mass tag
DEPs: Differentially expressed proteins
MOI: Multiplicity of infection
hpi: Hours post infection
PK15: Porcine kidney cells
TCID50: 50% Tissue culture infective dose
GO: Gene Ontology
KEGG: Kyoto Encyclopedia of Genes and Genomes
IBD: Inflammatory bowel disease
